# Multilevel barriers and facilitators to smoking cessation among men living with HIV in Vietnam: a qualitative study of male patients and healthcare providers

**DOI:** 10.1186/s12913-026-14087-z

**Published:** 2026-01-29

**Authors:** Thanh Ha-Lan Hoang, Claire V. T. Nguyen, Gloria Guevara Alvarez, Trang Nguyen, Nam Nguyen, Louise Adermark, Nawi Ng, Donna Shelley, Mari Armstrong-Hough

**Affiliations:** 1 School of Public Health and Community Medicine, Institute of Medicine, Sahlgrenska Academy, University of Gothenburg, Gothenburg, Sweden; 2 Department of Pharmacology, Institute of Neuroscience and Physiology, Sahlgrenska Academy, University of Gothenburg, Gothenburg, Sweden; 3 New York University School of Global Public Health, New York, NY USA; 4 Institute of Social and Medical Studies, Hanoi, Vietnam

**Keywords:** Smoking cessation, HIV, Implementation science, Tobacco use treatment, Barriers and facilitators

## Abstract

**Background:**

In Vietnam, tobacco smoking is highly prevalent among people living with HIV. However, research on engaging this population in tobacco use treatment is limited. To fill this gap, we aimed to identify barriers and facilitators to smoking cessation and examine implications for the design and implementation of tobacco use treatment for people living with HIV in Vietnam.

**Methods:**

We conducted in-depth interviews with 24 patients and 13 healthcare providers at three HIV outpatient clinics in Hanoi to explore barriers to and facilitators of smoking cessation at the patient-, provider-, and system levels. We conducted an abductive thematic analysis guided by the Theoretical Domains Framework.

**Results:**

Patient-level barriers to smoking cessation included patients’ perception that smoking is not an immediate threat to their health and does not impact HIV outcomes, lack of familiarity with cessation aids, stress, emotional distress, nicotine dependence, exposure to environments in which smoking is common, and social norms that promote male smoking and link smoking to masculinity. Patients and providers also described having “determination” to quit as an important precursor for making a quit attempt. Providers reported barriers to offering treatment, including lack of skills, knowledge, and competing demands related to treating tobacco use. However, they embraced their role in helping patients quit and described trusting relationships with patients as a potential facilitator of engaging smokers in cessation treatment. At the system level, both patients and providers identified easy access and affordability of tobacco products as barriers to cessation.

**Conclusion:**

This study identified opportunities for increasing engagement in tobacco use treatment at the patient, provider, and system levels. Provider training to deliver treatment, patient education about the health implications of continuing to smoke, system changes that support the integration of treatment into routine care, and revision of tobacco taxation are needed.

**Trial registration:**

ClinicalTrials.gov NCT05162911. Registered on December 16, 2021.

**Supplementary Information:**

The online version contains supplementary material available at 10.1186/s12913-026-14087-z.

## Background

The double burden of tobacco smoking and human immunodeficiency virus (HIV) transmission is a growing public health concern, especially in low- and middle-income countries (LMICs) [1, 2]. People living with HIV (PLWH) who smoke are at increased risk of cancer, pulmonary and cardiovascular diseases, and premature death compared with PLWH who do not smoke [3–5]. Smoking also increases the risk of opportunistic infections and developing acquired immunodeficiency syndrome (AIDS) in PLWH [6–10]. This double burden is especially pronounced in Vietnam, where smoking prevalence among men in the general population remains high and is even higher among men living with HIV (59% vs. 45%) [11, 12].

Vietnam has implemented several tobacco control measures since the enactment of the 2012 Law on Prevention and Control of Tobacco Harms, including taxation, smoke-free policies, advertising bans, and mass communication campaigns, in alignment with the WHO Framework Convention on Tobacco Control (FCTC) [13]. However, cessation support remains limited. Article 14 of the FCTC recommends that countries “take effective measures to promote cessation of tobacco use and adequate treatment for all tobacco users who wish to quit,” a mandate that applies equally to PLWH [14]. However, while solid evidence supports effective tobacco cessation interventions among the general population, research on specific approaches for PLWH is still limited [15–17]. Systematic reviews and meta-analyses show that clinician-delivered counseling and other interventions can yield short-term cessation benefits among PLWH [18–21], and the optimal strategies for integrating tobacco use treatment (TUT) this population remain unclear [22].

Most existing evidence on barriers and facilitators to quitting among PLWH originates from high-income countries [23]. The few qualitative studies from LMICs have not adequately explored how individual, social, and health system factors influence both patients’ smoking behaviors and healthcare providers’ readiness and capacity to deliver cessation support in HIV care contexts [24–26].

To generate contextually relevant evidence for policy and practice, this study was guided by established implementation and behavioral science frameworks. The Consolidated Framework for Implementation Research (CFIR) informed the exploration of system-level, organizational, and policy-related determinants influencing the integration of TUT into HIV care [27]. At the individual level, the Theoretical Domains Framework (TDF), which builds on the Capability, Opportunity, Motivation–Behaviour model, provided a structured lens for examining behavioral, cognitive, and social factors influencing smoking and cessation among PLWH [28, 29].

Guided by these frameworks, this study aimed to examine barriers and facilitators to smoking cessation among PLWH receiving care in HIV clinics in Hanoi, Vietnam, and further explored how healthcare providers envision supporting these patients to achieve smoking abstinence.

## Methods

### VQUIT study context – setting and design

This qualitative study was part of the formative research conducted prior to launching the VQUIT randomized controlled trial, which evaluated smoking cessation interventions for PLWH in Hanoi, Vietnam [30]. District Health Directors facilitated recruitment of outpatient clinics (OPCs) by contacting Medical Directors of the OPCs and coordinating communication with the research team. OPCs were eligible if they provided antiretroviral therapy to at least 240 patients at the time of enrolment in the trial. Three OPCs participated in this formative phase were based in District Health Centers, namely Hoang Mai, Dong Anh and Nam Tu Liem.

### Participant recruitment and enrollment for semi-structured interviews

#### Healthcare providers

At each participating OPC, research assistants (RAs) invited all healthcare providers (HCPs) to on-site informational meetings about the study, followed by scheduled interviews for data collection. Therefore, the number of HCPs invited per OPC is different for each site. The HCPs interested in the study were asked to sign consent to participate in the qualitative interviews.

#### Patients

**Fig. 1:**
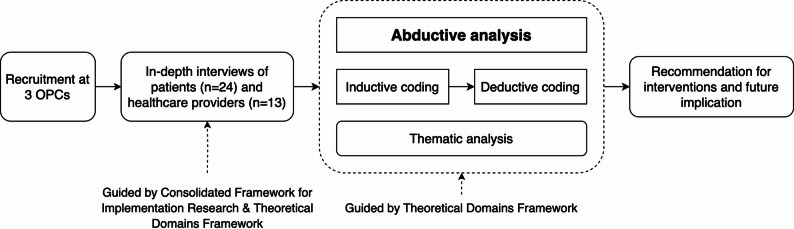
Study procedures

The nurses at the OPCs whose job it is to register patients when they arrive for their scheduled visit, received brief training on using the brief assessment tool that screened patients for tobacco use, on how to briefly describe the study, and to assess patients’ interest in learning more about the study. We used convenience sampling to select patients. The trained nurses identified patients who used tobacco as they checked in for their appointments. Patients who expressed interest in the study were directed to an on-site RA. The RA verified their eligibility, secured verbal consent, and arranged for interviews. We intended to recruit both male and female PLWH. However, due to the very low prevalence of smoking among women living with HIV in Hanoi, no eligible women were identified during the recruitment period. To qualify, patients needed to be at least 18 years old, own a mobile phone, smoke cigarettes daily or occasionally (with or without waterpipe use), and reside in Hanoi. In total 24 patients were screened, and they were all eligible for the study. None of them declined to participate. The study’s procedures are illustrated in Fig. 1.

## Interview guide development

We developed separate interview guides for HCPs and patients.

The HCP interview guide was informed by the CFIR and the TDF, which explored TUT-related knowledge and practice patterns, perceived capabilities to treat tobacco use, the feasibility and acceptability of integrating TUT into routine care, perceived relative priority of implementing TUT at OPCs, and perceptions about patient barriers to and facilitators of quitting smoking and engaging patients in TUT [27, 28].

The patient interview guide was informed by the TDF and explored current tobacco use patterns, perceived risks of smoking and benefits of quitting, quitting experience (including multilevel barriers and facilitators to quitting), social support, social influences, and community and workplace norms [28].

### Qualitative data collection

RAs worked for and were trained by the in-country organization that co-led this project (the Institute of Social and Medical Studies) and the New York University team. Specifically, they were trained to conduct qualitative interviews, including role-playing the interview guide. Four trained RAs conducted in-person semi-structured interviews with both patients and HCPs from November to December 2020.

#### Interviews with male PLWH

A total of 24 male PLWH were interviewed across three OPCs (OPC #1: *n* = 10; OPC #2: *n* = 6; OPC #3: *n* = 8). Interviews were conducted in private rooms at the clinics, lasted approximately 45–60 min, and were audio-recorded with consent. Field notes were taken during and after each interview.

#### Interviews with HCPs

Thirteen HCPs were interviewed at the same OPCs (OPC #1: *n* = 6; OPC #2: *n* = 4; OPC #3: *n* = 3). Provider interviews followed a separate guide but used the same procedures as patient interviews, including private settings, 45–60-minute duration, audio-recording with permission, and accompanying field notes.

All interviews were conducted in Vietnamese, transcribed verbatim, and translated into English. No repeat interviews were conducted, and transcripts were not returned to participants for member checking, consistent with local feasibility considerations.

### Data analysis

Two bilingual native speakers of Vietnamese with experience in qualitative methods (TH, CN) analyzed the transcripts using an abductive approach informed by the TDF and adapted from Thompson et al. [26]. In the first analysis stage, two research team members, TH and CN, listened to audio recordings, reviewed field notes and interview transcripts in Vietnamese and English, and used memoing to identify key themes and concepts. Independent line-by-line inductive coding was completed with five patient and three provider transcripts to generate an a priori code list. These codes were subsequently categorized within 12 TDF domains. All coding was conducted in Atlas.ti. and discussed at group meetings with the broader research team to generate a final code structure. The resulting codebook was used to code all patient and provider transcripts. Discrepancies were discussed and harmonized at group meetings with two other research team members (MAH, GGA). Tables S1 & S2 define the codes and the process for developing initial themes.

## Results

### Participant characteristics

Table 1 shows the characteristics of the participants. Twenty-four patients participated in interviews. Their median age was 44 (IQR: 40–47). Half of the patients reported using cigarettes only; the remaining half reported using both cigarettes and waterpipes. Among cigarette smokers, the median number of cigarettes smoked per day was 18 (IQR: 8–20). Among dual users, the median frequency of waterpipe use was 10 sessions per day (IQR: 7–12). Most were married, completed high school, and had annual incomes ranging from 100 to 300 million Vietnam Dong (approximately 4100 to 12 300 USD). Most patients reported that smoking in the home was allowed everywhere (*n* = 10) or in some places (*n* = 13). More than half (58%) of participating patients reported at least one past quit attempt.

Thirteen HCPs participated in interviews, including five physicians, five nurses, one pharmacist, one midwife, and one physician assistant. The median duration of working in the OPC was 10 years.

**Table 1:** Participant characteristics

Patient descriptors (*n* = 24)	*n* (%)
**Male**	24 (100.0)
**Age (Median [IQR])**	44 (40, 47)
**Age of smoking initiation (Median [IQR])**	17 (5, 18)
**Cigarettes per day (Median [IQR])**	18 (8, 20)
**Waterpipes per day in dual users (Median [IQR])**	10 (7, 12)
**Marital status**	
Married	14 (58.3)
Divorced	2 (8.3)
Separated	1 (4.2)
Single	7 (29.2)
**Types of tobacco use**	
Cigarette users	12 (50.0)
Dual users	12 (50.0)
**Ever made a quit attempt (abstain from smoking for at least 24 h)**	14 (58.3)
Levels of education	
High school and higher education	14 (58.3)
Lower than high school education	10 (41.7)
**Annual income (million VND)**	
< 10 m	1 (4.2)
10–50 m	3 (12.5)
50–100 m	9 (37.5)
100–300 m	10 (41.7)
300–500 m	1 (4.2)
Perceived Smoke-Free Home Rule*	
Allowed everywhere	10 (41.7)
Allowed in some places	13 (54.2)
Not allowed	1 (4.2)
**Provider descriptors (** ***n*** ** = 13)**	**n (%)**
**Age (Median [IQR])**	36 (30, 42)
**Length of work at OPC in years (Median [IQR])**	10 (2, 13)
**Gender**	
Male	1 (7.6)
Female	12 (92.3)
**Professions**	
Physician	4 (30.8)
Nurse	5 (38.5)
Pharmacist	2 (15.4)
Others (e.g., midwife, physician assistant)	2 (15.4)

*Patients were asked about whether tobacco smoking is allowed inside the home

### Main findings

**Fig. 2:**
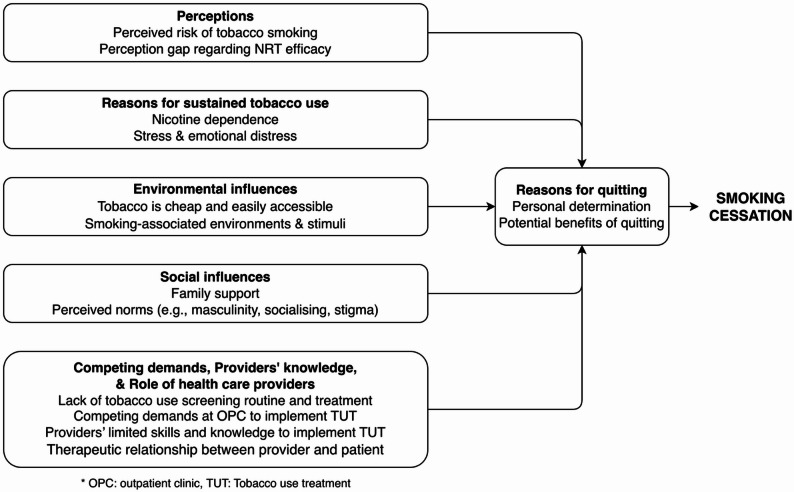
Diagram illustrating the determinants of smoking cessation in PLWH

Patient participants identified several potential barriers and facilitators to cessation (Fig. 2). They did not perceive smoking as an immediate threat to their health, as relevant to HIV outcomes, and did not think that treatment was available (*Perceptions*) (Table 2). They described living in environments in which smoking is common, normative, associated with masculinity, and highly accessible and affordable (*Environmental* and *Social Influences).* Providers also reported barriers to offering TUT, including their lack of skills, knowledge, and resources related to treating tobacco use (*Provider’s knowledge* and *Competing demands*). Both participant groups highlighted nicotine addiction and stress as major reasons for continued smoking. While patients mainly referred to stress, anxiety, or low mood, providers additionally noted that some patients’ emotional distress could contribute to challenges in quitting (*Reasons for sustained tobacco use*).

**Table 2:** Perceived barriers and facilitators to smoking cessation of smokers living with HIV and healthcare providers

Sub-themes	Themes and example excerpts	Barriers/ Facilitators
**Perceptions of Smoking Risk And Cessation Treatment**		
A. Patients’ perceptions about the risk of tobacco use	*“I haven’t thought of it yet. It may have an influence*,* but it hasn’t shown symptoms for me to avoid it actively. It still silently affects the internal body*,* so I haven’t thought deeply about the harmful effects of cigarettes*,* so I still smoke.” (Male cigarette user*,* married*,* age 40)* *“Either way*,* I will die. Then just take that [smoking] for fun.” (Male cigarette user*,* single*,* age 40)*	Barrier
B. Perceptions of smoking cessation treatment	*“If you give up smoking and then go buy those candies [NRT] every day*,* that cannot be considered a success.” (Male dual user*,* married*,* age 54)* *“[…] if it [NRT] worked*,* many people would quit. It’s not just me. If you could just use that patch and the gum to quit*,* many people would quit.” (Male cigarette user*,* married*,* age 57)* *“Because I have not been trained for the counselling on smoking cessation. My knowledge about it is vague. It is not deep enough.” (Female nurse*,* 10 years of work*,* age 38)*	Barrier
**Reasons for Quitting and Sustaining Smoking Behaviour**		
C. Reasons for quitting	**Perceived benefits of quitting** *“For example*,* when I quit smoking*,* it is good for my family if I stay at home and do not smoke. Then*,* it does not affect the children*,* my parents*,* the elderly.” (Male dual user*,* divorced*,* age 36)* *“… good for me. It [quitting] will reduce fatigue. Secondly*,* there are many things. It also reduces the [adverse] health effects. The next is less impactful on the finances.” (Male cigarette user*,* married*,* age 37)* **Personal determination** *“If I want to quit smoking*,* it will be up to my will and my own will only.” (Male cigarette user*,* single*,* age 42)* *“And if I am determined*,* there is no need for an influence from my wife like buying nicotine gum for me to help me reduce my cravings for cigarettes. It is my own will and determination that will help me quit smoking.” (Male cigarette user*,* married*,* age 40)*	Facilitator
D. Reasons for sustained tobacco use	**Nicotine dependence** *“In terms of quitting smoking*,* first*,* it would be the addiction*,* as we already know. Second*,* it would be the problem of not being determined yet. Third*,* during the process of quitting smoking*,* the patients may have some psychological pressure that makes them stressed*,* so they would want to smoke to relieve their mind*,* to be less pressured. That is all!” (Female physician assistant*,* one year of work*,* age 30)* *“Having been addicted for so long already makes me feel it is difficult to quit smoking by now. Quitting smoking for a day or two is quite easy. But after quitting for a day or two*,* I feel craving again. I feel very uncomfortable inside.” (Male cigarette user*,* single*,* age 42)* **Stress and Emotional distress** *“There are times when I feel kind of sad or happy*,* or depressed or cheerful*,* I often smoke as well. I also often smoke during those times.” (Male cigarette user*,* single*,* age 42)* *“To help me relieve stress a bit. For example*,* sometimes work is a bit stressful so … smoking a cigarette may make my mind a bit more comfortable*,* excited.” (Male dual user*,* married*,* age 44)*	Barrier
**Environmental Influences**		
E. Environmental cues to smoke	“*Basically*,* meeting my friends*,* sometimes going out to eat and karaoke*,* in that environment I can’t quit although I wanted to.” (Male cigarette user*,* married*,* 40 years)* *“Sometimes I manage to quit smoking once*,* or twice*,* but if someone sitting nearby smokes*,* I will smell the smoke and feel the craving […] when I went to work and had more [social] contact with my friends*,* well… when I kept seeing people smoking*,* I felt agitated*,* and so I relapsed into smoking.” (Male cigarette user*,* single*,* 42 years)*	Barrier
F. Accessibility and perceived low cost of tobacco	*“Because the money spent to buy tobacco is trivial and not a problem to say that it is a problem or such… not having money to buy [cigarette] so one quit*,* but tobacco is so cheap. Sometimes*,* strangers are asking you for a cigarette*,* you can still give them*,* for example.” (Male cigarette user*,* married*,* age 51)*	Barrier
**Social Influences**		
G. Family support	*“Generally*,* the family is important*,* mostly. Generally*,* relatives should give advice*,* remind*,* support*,* encourage*,* and so on. A bit of this and that so… reminds me so that I have the courage to quit.” (Male cigarette user*,* divorced*,* age 39)* *“In general*,* it [provider counselling] is good because when I understand more about cigarettes*,* I will realize it has no benefits. After that*,* I can be more determined.” (Male dual user*,* married*,* age 40)*	Facilitator
H. Social norms and masculinity	*“Men must drink alcohol*,* smoke waterpipe*,* and cigarettes. If a man does not drink alcohol or smoke cigarettes or waterpipe*,* he is a sissy. He is definitely a sissy.” (Male cigarette user*,* married*,* age 57)* *“There are times when you meet business partners*,* if they offer you cigarettes*,* you cannot refuse. It’s impolite and unsociable. You must take it when people ask. Some people do not smoke*,* but when they are offered a cigarette*,* they still have to take it. That’s it. It’s a must when socializing.” (Male cigarette user*,* married*,* age 51)*	Barrier
I. Discrimination and stigma	*“Because there are some patients… HIV patients who have very low self-esteem. They often overthink and have a complex about their conditions. They are afraid of being discriminated against… you know. So*,* at times like that*,* they only think of tobacco. So*,* their possibility of quitting smoking is also harder than that of common people.” (Female nurse*,* 2 years of work*,* age 25)* *“Receiving support [for smoking cessation for PLWH] from communes usually gets them discriminated. Hence*,* they do not want to let their communes*,* or families*,* know to support.” (Female physician*,* 10 years of work*,* age 66)*	Barrier
**Competing Demands**		
J. Competing clinical demands impede TUT	*“Not many of our staff would ask whether or not they smoked unless they had a cough or whatsoever. In here*,* we do not screen for tobacco use from the beginning. We just screen for tuberculosis.” (Female physician*,* 10 years of work*,* age 66)* *“Usually*,* I don’t… because patients are crowded*,* we cannot advise all the patients*,* but only the patients we meet and see they smoke a lot. The smell of tobacco is strong. Or if they smoke at this campus*,* I will remind them to reduce smoking […]. In general*,* there is also no intensive counselling for each smoker patient.” (Female pharmacist*,* 13 years of work*,* age 34)* *“For example*,* my health workers have scheduled appointments for seven patients. [ If they] have to spend time with two more smokers [for cessation counselling]*,* they will have to work overtime.” (Female physician*,* 15 years of work*,* age 36)*	Barriers
**Providers’ Knowledge**		
K. Providers lack knowledge about treating tobacco use	*“We have to go to training. If we were not trained*,* our confidence would not be enough [to provide counselling]. We have to go to training; we are only confident about ARV treatment and compliance counselling.” (Female pharmacist*,* 13 years of work*,* age 34)*	Barriers
**Role of Healthcare Providers in Treating Tobacco Use**		
L. Therapeutic relationship between provider and patient	*“Actually*,* the role of the health workers of the outpatient clinic in assisting patients in quitting smoking*,* if the activity is implemented*,* will be quite important because when the patients come here for follow-up examinations and are provided with counselling from the doctor*,* that counselling is received by the patients with trust and cooperation.” (Male physician*,* 2 years of work*,* age 46)* *“[…] So*,* gradually*,* the more I care about them… Well*,* when they feel such care matters*,* they will also… First*,* they will open their hearts. Second*,* they will be more friendly to us and accept to receive this counselling program.” (Female nurse*,* 2 years of work*,* age 25)*	Facilitator

* The Theoretical Domains Framework guides the organisation of this table

However, patients pointed to family and personal determination as potential facilitators of cessation (*Reason for quitting*, *Environmental* and *Social Influences)*. Similarly, providers were optimistic about integrating TUT into routine care delivery. Providers described strong, trusting relationships that could provide the foundation for engaging smokers in cessation treatment (*Role of health care providers*).

### Barriers to smoking cessation among PLWH

#### Patients’ perceptions about the risk of tobacco use

Patients were aware of the general harmful effects of tobacco and the benefits of quitting. However, ten did not perceive there to be a link between HIV prognosis and smoking (Table 2A).

No, there is no effects at all [of smoking on my current HIV condition] … It is not affected; cigarettes don’t have an effect. (Male dual user, married, 54)

One patient described switching to a waterpipe as a safer option when they learned that cigarettes were harmful to their health.

Later, when I knew that cigarettes were harmful to health, at the age of twenty, I switched to smoking waterpipe. Well, it was both less harmful and cheaper. (Male dual user, married, age 47)

Twenty-one patients expressed interest in quitting but believed they could delay this decision. Most perceived that in the short-term risk of tobacco-related illnesses was low and that other concerns were a higher priority (Table 2A). Two patients also expressed a sense of fatalism about their health, which was related to their HIV status. One of them noted: *“Either way*,* I will die. Then just take that [smoking] for fun.” (Male cigarettes only*,* single*,* age 40)*.

#### Perceptions of smoking cessation treatment

Thirteen patients were not aware that there were medications, like nicotine replacement therapy (NRT), to support smoking cessation. Those who had tried to quit described using non-nicotine gum, dried fruits, or candies to manage cravings when attempting to reduce or quit smoking. Two considered NRT to be another form of addiction and not effective as a cessation medication (Table 2B).

#### Stress and emotional distress

Both patients and providers described smoking as a coping mechanism for managing stress and low mood related to work and daily life pressures. Providers further noted that some patients also smoked to cope with broader emotional and social challenges, including perceived stigma, self-discrimination, unemployment, poverty, and family difficulties (Table 2D).

#### Nicotine dependence

Addiction to nicotine was described as a significant barrier to quitting (Table 2D): “Because I’m addicted, I can’t quit. Determining that I can never quit, why I have to quit or think of that.” (Male cigarette user, married, age 57).

#### Environmental cues to smoke

Patients described several environmental cues to smoking that impeded quit attempts. For instance, being around friends and family members who smoke, partying, and gatherings at public places like café, tea stalls, restaurants, and karaoke bars were major barriers to quitting. Participants also blamed smoking environments and cues for relapse following quit attempts (Table 2E).

The truth is that determination is a part. The second is the living environment. Now, if I am not in contact with the users, I think it will not induce craving when I successfully quit without further interaction with those people, right? (Male dual user, married, age 40)

#### Accessibility and perceived low cost of tobacco

Patients described tobacco products, particularly waterpipes, as inexpensive and perceived smoking as a behavior with little financial impact. Cigarettes and waterpipes are also easy to purchase at all convenience and grocery shops or street vendors (Table 2F). Although most patients were not motivated to quit by the cost of cigarettes, a few did note that saving money was a potential benefit of quitting smoking.

Because like that [my family’s economy will not be affected anymore when I quit smoking], I will contribute more income to my family. (Male dual user, married, age 40)

#### Social norms and masculinity

Patients cited strong social norms related to smoking as barriers to quitting (Table 2H). For example, patients associated smoking with masculinity: *“A man must drink alcohol and smoke. One surely needs both to be a real man. And if those who do not smoke*,* others will think that they are not men. Because it is also a cultural feature.” (Male cigarette user*,* single*,* age 40).*

Smoking is also still common among friends and colleagues of smokers.

As all my friends I hang out with are smokers, so I don’t know [how nonsmokers think about my smoking]. I don’t hang out with those nonsmokers, so I don’t know what they think. (Male cigarette user, married, age 51)

#### Discrimination and stigma

Besides self-discrimination and social stigma, providers described the concealment of HIV status as a barrier to quitting since some PLWH suffered from the internalized stigma that undermined social and family support in cessation (Table 2I).

#### Competing clinical demands impede TUT

Providers reported that they did not routinely ask about tobacco use or provide smoking cessation advice or counseling to smokers (Table 2J), citing a lack of training and a potential lack of time if they added this to their already busy schedules.

#### Providers lack knowledge about treating tobacco use

Health care providers described a lack of knowledge and skills to provide smoking cessation support (Table 2K): *“We have not had any knowledge on this topic yet. Currently, we have no knowledge at all.” (Female physician, 10 years of work, age 66).*

Providers expressed strong interest in helping patients quit smoking. Still, when the proposed intervention for the trial, which would require that they provide brief advice and counseling for smokers, they described having an already heavy workload and lack of training to provide the counseling.

Of course, the health workers will have to spend more time. They will have to be updated with new counseling skills. In general, there will be many difficulties when we participate in this. The health workers have to follow the patients closely so that the patients will come back. (Male physician, 2 years of work, age 46)

### Facilitators of smoking cessation among PLWH

#### Perceived benefits of quitting

Patients reported efforts to quit in response to concerns about their health and the health of family members, especially for children, older people, and the community. Besides health benefits, they also mentioned potential financial gains as money used to buy tobacco products was saved for other expenses (Table 2C).

Commonly cited reasons for temporary quitting included having a severe illness that prevented them from smoking. However, patients reported that they started smoking again when their health was improved.

I have just reduced it only. I have never thought about quitting smoking completely, only reducing smoking. So, when my body is back to normal, I will smoke again. (Male dual user, married, age 46)

#### Personal determination

Patients frequently emphasized personal determination as vital for motivating and sustaining a quit attempt (Table 2D).

In my opinion, right now, the most important thing about quitting smoking is one’s determination. (Male dual user, single, age 39)

Even though patients acknowledged the influence of support from family and providers, awareness of smoking’s harms, and severe illness on their decision to quit, they believed that determination alone was sufficient for success.

[…] And if I am determined, there is no need for an impact from my wife like buying nicotine gum for me to help me reduce my cravings for cigarettes. It is my own will and determination that will help me quit smoking. If I just rely on my wife’s words like: “Please quit smoking, I will buy you gums”, I don’t think it will work. Mainly my will, determination, to quit smoking. (Male cigarette user, married, age 40)

Health care providers used similar terminology when describing barriers to quitting. “I can only support them mentally. If patients do not have their determination, it is very difficult to quit” (Female physician, one year of work, age 50).

#### Changing environmental cues

Patients reported that refraining from smoking in smoke-free public spaces and avoiding smoking environments could aid quitting. Some mentioned strict COVID-19 lockdowns, stays in rehabilitation centers, and hospitalization as situations in which they stopped smoking.

The reason was hospitalization, due to an accident, so that … I did not get much contact with the people (outside of the hospital). Hence, my smoking was also limited. (Male cigarette user, single, age 40)

#### Family support

Patients valued family encouragement as a motivation to quit, often characterized by gentle persuasion, well-intentioned pressure, and emotional support (Table 2G). Health care providers also described family support as essential to facilitate cessation:

Quitting smoking also requires the support of their family. So, as I said… Actually, I could make some impact on them. However, for the patient, there are other factors the family must support. (Female nurse, 12 years of work, age 36)

Nonetheless, patients emphasized that while family members offered significant support and advice, the decision to quit smoking rested with the individual.

Support? To be honest, whether I smoke or not is all up to me. The determination is on me. Otherwise, if someone wants [to support], like my wife at best, she will buy me the mouthwash to rinse my mouth, so I do not have a craving when I do not smoke. But mostly, it is on me. (Male cigarette user, married, age 46)

#### Therapeutic relationship between provider and patient

Health care providers endorsed their vital role in providing TUT and building trust in patient-provider relationships, which could facilitate patient adoption and adherence to potential TUT (Table 2L).

[…] So, gradually, the more I care about them… Well, when they feel such care matters, they will also… First, they will open their hearts. Second, they will be more friendly to us and accept to receive this counseling program. (Female nurse, 2 years of work, age 25)

## Discussion

This qualitative analysis found that barriers to quitting included perceptions that smoking is inexpensive, persistent social norms that link smoking to masculinity, and the belief that smoking is not impacting HIV-related outcomes. Family support and personal motivation to quit emerged as facilitators to the uptake of TUT.

The finding that PLWH who smoked did not perceive smoking to be particularly risky is consistent with prior findings that patients lack knowledge about how smoking can negatively impact HIV outcomes despite having a general awareness of smoking harms [24, 31]. Both patients and providers reported limited knowledge about the availability or effectiveness of smoking cessation treatment. These findings highlight the need for targeted education and awareness campaigns aimed at both PLWH who smoke and their healthcare providers that provide evidence-based information about the specific risks of smoking for PLWH and the available smoking cessation treatments.

Despite implementing the full range WHO-recommended tobacco control policies, including smoke free air laws, social norms in Vietnam that link smoking with masculinity and social bonding continue to create pressure for men to smoke in social situations [12, 23, 32, 33]. This is phenomenon is similarly reported in countries with smoking rates that are disproportionately higher among men compared with women [34–39].

However, there is evidence that in Vietnam, population norms are changing. For example, participants acknowledged that nonsmokers may be bothered by exposure to secondhand smoke (SHS) [13]. A systematic review and meta-analysis of Global Adult Tobacco Survey (GATS) data reported that household secondhand smoke exposure in Vietnam declined from 49% in 2010 to 37% in 2015 [40]. However, SHS exposure remains high (> 80%) in bars, cafés, tea shops, and restaurants [41], which PLWH in our study described as smoking-associated environments. A recent qualitative study among a general population of male smokers in Vietnam suggests that altering normative behaviours related to male tobacco use may be influenced through more consistent implementation of tobacco control polices such as enforcing smoke free air policies, increasing the price of tobacco and greater counteradvertising [33].

All smokers in our study reported that tobacco is cheap and easy to purchase, which could be due to Vietnam’s low cigarette tax rate compared to other countries in the Western Pacific Region [42, 43]. The ad valorem tax rate in Vietnam is only 35.6% of the retail price, lower than the WHO’s recommended 70% and the regional average of 55.6% [42, 44]. Besides, household incomes in Vietnam have increased significantly by more than 500% between 1994 and 2017, while the price of cigarettes only rose by a small amount of 6% during this period [45, 46]. This means that cigarettes have become much more affordable for Vietnamese people over time. A pack of cigarettes typically costs around 15,000 to 20,000 Vietnamese dong (VND), approximately 0.59 to 0.79 US dollars (USD) [46, 47]. In addition, research consistently shows that lower tobacco retailer density and increased distance from retailers are linked to reduced tobacco use [48]. Alongside a substantial cigarette tax increase, limiting the number and proximity of tobacco retailers may help decrease consumption [49, 50]. Still, further research is needed to evaluate the impact of such policies in Vietnam.

Our interviewees also described smoking addiction, emotional distress, and stress as major barriers to quitting smoking, reflecting a complex interplay of factors unique to this population. The co-occurrence of smoking and depression among PLWH has been widely reported in the literature [21]. Moreover, PLWH with a history of depression are less likely to sustain smoking abstinence [51, 52]. The prevalence of depressive symptoms among smokers living with HIV in Vietnam was high (38.3%) [53]. Studies confirmed our findings that PLWH are more likely to experience emotional distress due to the HIV-associated stigma [54, 55]. These factors, combined with the physiological interactions between HIV, antiretroviral therapy, and nicotine metabolism, further result in enhanced nicotine dependence and complicate cessation efforts [56]. The consistent findings that stigma, emotional distress, and stress are impeding smoking cessation also require research on approaches that specifically intervene to address these significant barriers. These findings underline the importance of integrating mental health support into smoking cessation programs for PLWH. Addressing depressive symptoms and stress as part of comprehensive tobacco cessation interventions could improve outcomes.

Smokers and healthcare providers in our study frequently used the term “determination” to describe the personal motivation or commitment they saw as necessary to consider a quit attempt. Other studies among Asian populations, such as among Chinese and Vietnamese men living in the US, similarly found that willpower or determination was perceived as an essential facilitator to quit smoking, despite the broader availability of smoking cessation services that offer smokers alternatives to depending on internal motivation [31, 57, 58]. Considering the role of Self-determination Theory (SDT) is interesting in this context [59]. SDT suggests that fulfilling the basic psychological needs of autonomy, competence, and relatedness enhances intrinsic motivation. According to SDT, internalization shifts motivation from extrinsic to intrinsic, promoting behaviour change [59]. Although SDT-based interventions for smokers with HIV are limited, they have been effective for other groups, increasing quit rates and treatment use by addressing autonomous interest and self-efficacy [60–67]. Given the lack of long-term effective interventions for PLWH who smoke, exploring psychosocial approaches, including those based on SDT, is essential. Aligning interventions with these psychological needs can foster sustainable motivation to quit smoking among PLWH, especially where service access is limited and personal determination is culturally important.

Healthcare providers in our study similarly described barriers to adopting TUT in routine practice, including competing demands. The implementation of smoking cessation programs in community health settings has been hindered by the demanding schedules of healthcare providers, with many prioritizing other tasks, especially in LMICs, where tobacco cessation is viewed as non-urgent [68–71]. In Vietnam, community health workers often face barriers to delivering smoking cessation services, such as time constraints, lack of training, and low national priority [72]. Nevertheless, providers in our study believed they had an important role to play in improving their patients’ access to treatment, and patients were interested in obtaining support from their HIV clinicians to quit smoking. This indicates an opportunity to integrate tobacco cessation services into HIV care. However, there is a lack of strategies for such integration. HIV clinic staff lack training and resources for tailored cessation interventions [24, 73]. Therefore, it’s crucial to develop and evaluate tailored cessation interventions for PLWH, train HIV clinic staff on tobacco cessation, and allocate resources for integration, including systematic screening, counselling, and access to cessation medications.

There were several limitations to this study. First, we did not interview women living with HIV who smoked because we were unable to sample a sufficient number. The prevalence of smoking among women in Vietnam is very low. Second, we recruited participants from three OPCs in Hanoi, Vietnam. Therefore, findings may not be generalizable to other contexts or regions. However, the Hanoi experience may apply to similar urban settings in Vietnam or other countries with comparable sociocultural characteristics. The strengths are the rigorous data analysis process and triangulation of respondents, and the use of two determinant frameworks to guide data collection and analysis.

## Conclusion

High rates of tobacco use among PLWH in Vietnam and other LMICs require that health systems develop strategies for integrating TUT in the context of HIV care. This study adds to the growing literature on factors influencing tobacco use and barriers to quitting in this population. Many of these barriers can be addressed by leveraging the HIV care system infrastructure to scale provider training and system changes that support routine screening and treatment of tobacco use and other substance use disorders. Additional research is needed to study cessation interventions that address barriers to quitting that are specific to this population, including stigma and high rates of mental distress. Still, the need for further advances in treatment approaches should not preclude implementing WHO recommendations to close the global health equity gap for smoking PLWH.

## Supplementary Information

Below is the link to the electronic supplementary material.

Supplementary Material 1

Supplementary Material 2

## Data Availability

The datasets are available from the corresponding author upon reasonable request.

## Abbreviations

CFIR: Consolidated Framework for Implementation Research
GATS: Global Adult Tobacco Survey
HCP: Healthcare provider
HIV: Human immunodeficiency virus
LMIC: Low- and middle-income country
NRT: Nicotine replacement therapy
OPC: Outpatient clinic
PLWH: People living with HIV
SDT: Self-determination theory
SHS: Secondhand smoke
TDF: Theoretical domains framework
TUT: Tobacco use treatment
WHO: World Health Organisation
