# Supplementary material for: Multilevel barriers and facilitators to smoking cessation among men living with HIV in Vietnam: a qualitative study of male patients and healthcare providers
Source: BMC Health Serv Res. 2026 Jan 29;26:312. doi: 10.1186/s12913-026-14087-z (PMC12937535; doi:10.1186/s12913-026-14087-z)
Supplement: Supplementary file 1 — Supplementary Material 1 [file 12913_2026_14087_MOESM1_ESM.pdf]

Patient individual interview guide

Date: _____/_______/_______**Interviewer initials**: _____

Interview location (circle one): In person   Telephone

Time Start: _______________   **Time stop:** ________________

Participate code: ________________

(Notes in these parentheses are for the moderator – not to be read out loud.)

Materials need onsite

- *Pens/Paper*
- *Digital recorders (2)*
- *A nicotine patch, a box of chewing gum OH NO to show during QUITTING EXPERIENCE section*

Introduction (3 minutes)

Hello.
My name is _*[first name]* and I am from the Institute of Social and Medical Studies.
Thank you for your willingness to take part in this interview. The purpose of this discussion is to hear your views about smoking, challenges you have experienced if you have tried to quit, reasons why you do and don't want to quit smoking and what types of programs or services you think would help you quit. Your ideas are very important to us and I appreciate that you have taken the time to talk to me today.

We will have about 45-50 minutes for our discussion. To ensure that we are able to capture accurate and complete responses, we would like to record this interview. The recording will be transcribed, and all names and places will be removed so as to protect your identity. Your personal identification information such as your name will not be included in the transcripts

I want to remind you that everything we discuss today will be confidential. We are not asking you to disclose any personal information. No one will hear this recording except for people working on the project. Whenever we write a report, we will describe our findings in a way in which no one can identify you. If there are any questions you do not want to answer, just let me know - that's fine. Your participation is completely voluntary. There are also no right or wrong answers. We want to know your opinion and what you think about the issues we will be discussing.

Do you have any questions before we begin?

**A-ICE BREAKER**	1. Can you tell me about how you started to smoke cigarette/waterpipe? *Probe: when, why, with whom*?<br>2. When are you most likely to want to smoke or to crave a cigarette/waterpipe? *Probe: can you give me examples of situations or people or mood that trigger your smoking?* [if dual user, ask this for both cigarettes and waterpipe]<br>3. Where to you usually buy cigarettes/waterpipe*? **Probe**: what type of store, from friends, online*<br>4. How do you usually buy them, in a pack, carton, single cigarette or in another way? How often do you buy? [if dual user, ask this for both cigarettes and waterpipe]<br>5. How does your spending for smoking impact your ability to spend money on other things you need?
**B-BARRIERS TO QUITTING**	I'd like to turn to talking about your smoking history and experience.<br>*1.* First, can you share with me what you like about smoking? ***Probe:*** *reduces stress, relieves boredom, anxiety.*<br>2. What don't you like about smoking? ***Probe***: *cost? smell on your clothes, impact on family, discrimination from nonsmokers?*<br>3. Its sounds like there are some good things and bad things about smoking. Why do you think you continue to smoke? Probe: smoking helps with stress, anxiety, depression, deal with the disease (HIV); is a social activity; gives energy and pleasure; too hard to quit
**C-HEALTH EFFECTS OF SMOKING**	How do you think smoking is affecting your health? ***Probe***: *What about the impact on your HIV infection? How do you think smoking may affect your risk of getting TB? How concerned are you about these risks?*<br>*(NOTE to facilitator: Be prepared to briefly answer questions about how smoking effects HIV and to probe then what they think about that information)*
**D-PERCIEVED BENEFITS OF QUITTING**	1. I'd like to ask you some questions about quitting smoking. What are some reasons that smokers you know have quit?<br>2. What would make you decide to quit smoking?<br>3. What do you think would be the benefits of quitting? ***Probe***: *better health, family health, more money*
**E-SOCIAL INFLUENCES (5min)**	1. What does your family think about your smoking? ***Probe: trying*** to get you to quit, do they smoke with you?<br>2. What do your friends who are non-smokers think about your smoking? How about friends who are smokers?<br>3. In what social situations do you smoke? ***Probe***: with friends, parties, bars, probe if family and friends are smoking in those situations<br>4. When you are in these social situations how would you feel if you weren't smoking? ***Probe:*** *out of place, stressed, pressured to smoke*<br>5. What could your family and friends do that would help you quit? What kind of support could they provide? ***Probe:*** *Can you share an example of how you think family or friends could support you to quit smoking?*
**F-QUITTING EXPERIENCE**	1. Have you ever tried to quit?<br>**a.1**. **If response is NO**, ask: What would motivate you to try to quit? (NOTE TO FACILIATOR: may have been answered under benefits of quitting section)

	**a.2.If response is YES**, ask: Think about the last time you tried to quit, can you describe your experience? What was that like?<br>***Probe****: when, why did you quit? What happened? What was difficult about trying to quit? What worked? What didn't work?*<br>2. There are also medications like nicotine patches, nicotine chewing gums that can help people quit smoking by reducing cravings. Can you tell me what you know about these medication? ***Probe****:* Have you used these medications? *why, why not and experience with them-were they helpful or not)*<br>3. For those who have not tried to quit, can you share why you haven't you tried?
The next questions will ask you what you think about some ways in which smokers could get help quitting.	
**G-TOBACCO CESSATION PROGRAMS**	1. What do you think is the best way/support to help smokers quit? ***Probe****: counseling, workshops, medication, other support, what would help you?*<br>2. How would you feel about having your doctor or nurse talk to you about quitting? ***Probe****: how can they be helpful?*<br>3. There is a national telephone counseling program referred to as a Quitline that is free of charge. You can call the program to get help from a trained smoking cessation counselor.<br>What do you think about calling the Quitline to get help? ***Probe****: Do you think you would call? If No, Why? What would motivate you to call them? How do you feel about your health providers connecting to the Quitline for you during your visit?*
**H-REACTION TO PROPOSED NURSING COUNSELING INTERVENTION**<br><br>*Now I'd like to know what you think about a program that is designed to help smokers quit.*	*Our idea is to train nurses in your clinic so that they are comfortable speaking to smokers in a way that may encourage them to quit. The nurse would offer 6 sessions of counseling over a 3-month period. These sessions would be in person and by telephone and last about 30-45 minutes.*<br>*So imagine that you are coming into the clinic to get your medication and you are approached by a nurse and asked if you would be willing to participate in a counseling program with the first session taking place at that visit.*<br>**1.** Do you think you would participate in this program and receive 6 sessions of counseling? ***Probe****: Why, or why not, what would make you participate? What do you like about the idea? What don't you like?*<br>***2.*** *What difficulties you expect to face when participating? How to overcome these? How do you think about number of counseling sessions and length of sessions? What day of the week do you want to receive intensive counseling sessions? How about weekend? What time of the day do you want to receive intensive counseling?*<br>***3.*** How do you feel about receiving counseling by phone as compared to in person? ***Probe****: advantages, disadvantages*<br><br>*We are also considering sending text messages to help smokers quit. These would be sent a couple of times a day and include supportive statements and helpful ideas for avoiding smoking and supportive messages. For example, a message might say:*<br><br>***"Next time you have the urge to smoke, strengthen your willpower by trying to resist for 5 minutes. The craving will pass. Think of it as practice for quit day!"***

	I'd like you to think about what you are doing, for example, in the morning at 9 or 10 o'clock and imagine receiving a message like this.<br><br>4. How would that feel? ***Probe**: do you think you would want to receive it? If No, Why? what messages/contents could be helpful? When in the day the messages should be sent? Number of messages a day?*<br>5. What do you think about combining this with counseling? So for example, imagine you are participating in the counseling program and in between sessions you receive these messages? What do you think about that idea? *(**Probe**: helpful to get the extra support, or disruptive?*
	Thank you all for your time and willingness to speak with me today.

List of probes of reasons for smoking

Pleasurable
**Alleviates boredom**
**Helps with stress / it's relaxing**
**Manages depression**
**Manages anxiety**
**Manages anger**
Avoidance of withdrawal symptoms
**Helps pain meds work better**
**Helps HIV medication work better**
Helps remove the bad taste from medications
Smoking helps me deal with my HIV infection
No motivation to quit
It's a social activity; many friends or family members smoke
Keeps my weight down
Addicted to it
It keeps me from doing harder drugs/alcohol
It gives me energy
Helps me concentrate better
Too hard to quit
Never received medical advice to quit
Not worried about my health

Health Care Provider Interview Guide

Hello.
My name is ___________[first name] and I am from the Institute of Social and Medical Studies.
Thank you for your willingness to take part in this interview.

We are conducting a study to evaluate the effect of a smoking cessation program designed for HIV positive tobacco users. The purpose of this discussion is to hear your views about what challenges smokers are experiencing that may make it difficult to quit using tobacco. We are interested in any experience you may have had in supporting smokers to quit, your experience with patients who use tobacco, and to learn more from you about how we can help these patients quit. We would also like to get your feedback on the program we are proposing. Your ideas are important to us and I appreciate that you have taken the time to talk to me today.

We will have about 45-60 minutes for our discussion. To ensure that we can capture accurate and complete responses, we would like to record this interview. The recording will be transcribed, and all names and places will be removed to protect your identity. Your personal identification information such as your name will not be included in the transcripts.

I want to remind you that everything we discuss today will be confidential. If there are any questions you do not want to answer, just let me know - that is fine. Your participation is completely voluntary. There are also no right or wrong answers. We want to know your opinion and what you think about the issues we will be discussing. Your participation is voluntary.

Do you have additional questions? Do I have your permission to start the recorder?
I'd like to start by asking what your role is in the OPC.

MAIN TOPICS	QUESTIONS

**A. Role of health care worker**<br><br>***5 minutes***	Head of OPC:<br>Can you describe briefly functions of the OPC? Target? Open time, weekday, weekend?<br>Can you tell me about the OPC personnel? Positions and roles of OPC staff? What are your responsibilities?<br>All other Health providers:<br>I'd like to start by asking you what are you position and your role is in the OPC?<br>***Probe:*** *specific responsibilities*
**B. General questions about OPC services**<br><br>***10-15 minutes***<br><br>NOTE: Medical director at each site will be asked B1 and note taker will document the patient flow based on responses and then show the diagram to the MD for feedback. Based on feedback, the diagram will be revised until MD confirms that this is accurate. Interviews with other HCWs will include showing the staff the workflow diagram and asking them to provide feedback. Interviewer will document all recommended changes on the diagram	1. a) Head of OPC: Can you describe a typical patient visit, for old patients with follow up visits? ***Probes***: For example, what happens when a patient comes into the clinic for their visit? Who do they see first? How is the visit documented? Who does testing and counseling? When does that happen during the visit?<br>On average, how many patients visit the OPC a day? Which days are there more patients? How about morning compared with afternoon?<br>b) Other health providers: [Interviewer shows the patient flow diagram and ask]<br>Here is the patient flow diagram, do you want to change or add any things?<br>2. How do you screen patients for TB? How do you screen patients for mental health problems?<br>3. What services that the OPC does not provide so you need to refer patients? how about drug use, TB, and mental health? ***Probe:*** where do you refer patients to? How do you make the referral? How do you follow up?<br><br>5. Can you tell me about how new OPC programs are implemented at this clinic? ***Probe****:* What does senior leadership do? How about providers?<br>6. When a change needs to be made to a program, how does the OPC make the change? Prob: Who decides the change? How to make the change?<br>*7.* What are the biggest challenges you face in implementing the OPC's programs? ***Probe*****:** infrastructure, equipment**,** *lack of training, not enough staff, need for computers/IT,* patient resistance, lack of time<br>*8.* What is the support needed to implement a new program? Probe: Leader's support, infrastructure, finance...How important is support from the leadership at the District Health Center and Provincial department health leadership in being able to implement new programs? ***Probe:*** Why did you say that?
Now I'd like to ask you about your experience with patients who smoke	
**C. Knowledge and Beliefs about tobacco use among HIV patients and barriers to quitting**<br><br>***10 minutes***	1. What do you think are the health risks for your HIV infected patients who smoke compared with those who don't smoke?<br>2. Do you notice any differences between patients who smoke and those who don't smoke in terms of their health?<br>***Probe****: IF ANSWER NO-probe differences in number of infections, differences in adherence to ART*<br>***Probe****: IF YES, probe what types of differences*

	3. **What do you think are the challenges HIV+ smokers experience when trying to quit? *Probe*:** social pressure, lack of support, stress, depression (see appendix reasons smoke)<br>4. **What do you think would help H+ smokers quit?** Probe: motivation for health and family's health, self-determine, family support...<br>***Probe:*** *Can you share an experience with a patient who did try to quit smoking and was successful or one that wasn't and why**?***
**D. Role and Relative Priority of tobacco use treatment**<br><br>***5 minutes***	1. Relative to other program/activities, how important do you think it is to help smokers quit? ***Probe*:** *why?*<br>a. What about among leadership at the district and provincial level?<br>b. How important is tobacco use relative to other programs?<br>2. What is your role in terms of helping smokers quit? ***Probe:*** *Do you view this as part of your role? If not, who should be helping smokers quit?*
**E. OPC polices and providers' current practice**<br><br>**5 minutes**	1. How often to you ask patients about tobacco use? ***Probe***: every visit, only at first visit, never<br>2. How do you feel about offering smokers cessation counseling or other help to quit? ***Probe***: How confident are you that you can help smokers quit? Why?<br>3. Does the OPC have a policy for identifying smokers? If Yes, what is that? ***Probe:*** are you expected to screen for tobacco use and offer counselling and treatment?
**F. Feedback on program and program fit**<br><br>**[Interviewer show the intervention diagram and describe the intervention and ask if they have clarifying questions]**.<br><br>**20 minutes**	[**Interviewer:** Have the revised patient flow diagram as a guide during this section. The interviewer can ask where in the diagram they think there could be barriers to implementing the program].<br>1. Have you or the OPCs implemented anything like this program before? If Yes, what were that? ***Probe*: i**ntervention programs related to smoking cessation, other programs, *Experience with referring patients to the Quitline?*<br>2. How will asking patients about tobacco use and providing brief smoking cessation counseling fit into your current workload? ***Probe***: *Is it feasible, why, why not, will it take too much time*<br>3. [interviewer shows the patient flow chart] When during the patient visit would the screening and brief counseling be done? Who should do this? Why? ***Probe:*** *think about the patient visit flow.*<br>4. What challenges do you anticipate in including screening for tobacco use and brief counseling patients during patients' visits? ***Probe****: lack of time, competing demands, patient resistance, lack of training and knowledge about tobacco use treatment, patients have more important issues to deal with, What else?*<br>5. [interviewer shows the patient flow chart] What about referring patients to the Quitline? How would this fit into the patient visit? ***Probe:*** *When could this happen during the visit?*<br>6. How do you think patients will react if you recommend that they call the Quitline? ***Probe****: receptive, suspicious, privacy concerns, not interested in quitting. Do you think they will call the Quitline? Why?*

	7. What do you think would encourage smokers to call the Quitline? What could the OPC or you do to increase the likelihood that they will call? How can you help the patient call the Quitline? Can you call the Quitline for the patient during the visit and connect them directly to the counselor? Who could do that in the clinic?<br>8. Who should be the ones who conduct intensive counseling for smokers? What about nurses? How does that role fit with current responsibilities?<br><br>Is the nurse the right person to train to offer more intensive counseling to patients who smoke? ***Probe:*** *are there other staff who might be trained to do this?*<br><br>[**NOTE:** if interviewing nurses we want to know how they think this role fits in current workload with probes as above]<br><br>Some patients will not be able to come in for in person counseling and will need to have some counseling sessions by telephone.<br>When would you be able to do telephone calls? What time of day would be most feasible?<br>9. What do you think would encourage smokers to agree to get support by attending multiple counseling sessions from a nurse or other staff?<br>a. What do you think would motivate them to attend all of the 6 sessions of counseling? (Making some of them phone calls, incentives)<br>b. What can you do to encourage patients to enroll in counseling?<br><br>10. What challenges will patients face if they participate in the program? ***Probe:*** *challenges attending all of the counseling sessions, challenges quitting*<br><br>11. What challenges will health providers face in implementing the intensive counseling to patients? Probes: *lack of time, competing demands, patient resistance, lack of training and knowledge about tobacco use treatment, patients have more important issues to deal with, What else?*<br><br>12. What support does the OPC need to implement this program? ***Probe:*** *MOH, other leadership? Resources, support for health providers, support for patients.*<br><br>What support do you need to help your patients quit? Probe: materials, money incentives for patients.<br><br>13. Overall, how confident are you that the OPC can implement tobacco use treatment as part of routine care? ***Probe:*** Why, why not?
**G. Competing priorities**<br><br>***5 minutes***	1. What other important programs or events are ongoing that may make it hard for health care providers to devote time to offering smoking cessation support? ***Probe:*** *How do you think those will affect this program?*<br><br>2. What other initiatives, programs, or activities are planned that would support this program? ***Probe****:* *For example, other prevention or public health programs, or other tobacco control programs?*

Description of the program

[**The interviewer** shows the diagram depicting the program with three models (with more details about the number of patients, number of counseling sessions, and timeline...)]

As we mentioned at the start of the interview, we are developing a program to help smokers quit. We are evaluating three versions of the program. That means that during the study, not all patients will receive all the components. 48 patients will be randomly assigned to one of the three versions, 16 for each version. It is important to evaluate each one to see which combination works best. I am going to describe the three versions and ask for your feedback.
For the first version, we will train providers in your OPC to screen all patients for tobacco use, offer brief counseling to those who smoke and refer smokers to the national Quitline. The Quitline provides free support delivered by trained counsellors to help smokers quit cigarettes and waterpipe. Providers will have brochures to give to smokers that will have information about the health effects of tobacco use and the Quitline number.
The second version of the program adds a referral to a trained nurse in the OPC who will provide 6 sessions of tobacco cessation counseling over a 3-month period.
The third version includes everything in version one and 2 but also adds 4 weeks of free nicotine replacement therapy.
