# Supplementary material for: Multilevel barriers and facilitators to smoking cessation among men living with HIV in Vietnam: a qualitative study of male patients and healthcare providers
Source: BMC Health Serv Res. 2026 Jan 29;26:312. doi: 10.1186/s12913-026-14087-z (PMC12937535; doi:10.1186/s12913-026-14087-z)
Supplement: Supplementary file 2 — Supplementary Material 2 [file 12913_2026_14087_MOESM2_ESM.docx]

**Table S1. Development of initial themes relating to barriers and facilitators of smoking cessation in PLWH perceived by both patients and providers at OPCs**

| **TDF domains** | **Patient interviews** | | **Provider interviews** | | **Initial themes** | **Barrier/ Facilitator** |
| --- | --- | --- | --- | --- | --- | --- |
|  | **Codes** | **Description** | **Codes** | **Description** |  |  |
| **Knowledge** | Knowledge about smoking and HIV | Limited knowledge of the smoking impact on HIV condition; Still healthy as no manifestation of diseases; Dying from AIDS anyway | Knowledge about smoking and HIV | Patients have yet to understand the impact of smoking harms | Patients’ perceived knowledge about smoking harms and TUT | **Barrier** |
|  | Knowledge about quitting smoking | Not aware of smoking cessation support; Doubting the effectiveness of NRT |  |  |  | **Barrier** |
|  |  |  | Knowledge about TUT | Providers are untrained to provide TUT | Providers lack the knowledge to perform TUT counselling | **Barrier** |
| **Skills** | Skill to quit smoking | No experience with using NRT to quit; Using normal gums/candies to reduce cravings |  |  | Unawareness of NRTs | **Barrier** |
|  |  |  | Skill to provide TUT | Providers are untrained to provide TUT | Providers have limited skills in performing TUT counselling | **Barrier** |
| **Beliefs about capabilities** | Pessimism about the ability to quit | Quitting is difficult; Pessimistic about the ability to quit | Pessimism about patients’ ability to quit | Pessimistic about patients’ ability to quit | Perceived poor self-efficacy to quit | **Barrier** |
|  |  |  | Lack of confidence to provide TUT | Not confident to implement TUT | Providers’ perceived lack of confidence in implementing TUT | **Barrier** |
|  |  |  | Pessimism about patients’ interest in quitting | Anticipated poor patient participation in TUT; Lack of patients’ interest in TUT | Pessimism about patient participation in TUT | **Barrier** |
| **Beliefs about consequences** | Quitting benefits  Smoking consequences  Rewards for quitting | Quitting benefits for finance and health of patients, family, and others; Smoking impacts finance and health of patients, family, and others; Rewards and incentives from family if quitting smoking | Quitting benefits for patients | Patients quit to improve health and finance | Extrinsic motivation to quit: Health and financial improvement | **Facilitator** |
| **Motivation & Goals** | Determination/willpower | “Quitting or not is up to me”; Determination to quit smoking | Determination/willpower | Determination (or willpower) is needed to quit smoking | Lack of determination as the motivation to quit | **Facilitator** |
|  | Intention to quit | No intention to quit | Patient’s intention to quit | Patients’ intention to quit determines their participation in TUT | Mixed intention to quit in patients | **Barrier or Facilitator** |
| **Environmental context** | Cheap tobacco  Easy to buy | Cheap cigarettes & waterpipes; Tobacco inviting culture; Can buy tobacco easily | Cheap tobacco | Cheap tobacco products | Affordable & widely accessible tobacco products | **Barrier** |
|  | Smoking stimuli  Smoking environment | Seeing someone smoking; Smokers in family/close relationship; Smoking stimuli (e.g., drinking tea, coffee, and alcohol; gatherings; boredom; stress/depression; after meals/waking up, etc.) | Smoking stimuli  Smoking environment | Smoking environment; Smoking stimuli (e.g., drinking, gatherings, and substance use) | Smoking environment, drinking habit, substance use, and other stressors hindering smoking cessation | **Barrier** |
|  |  |  | Availability of tobacco use screening and treatment | No screening for smoking; No tobacco use intervention | No tobacco use assessment and intervention at OPCs | **Barrier** |
|  |  |  | Limited infrastructure  Lack of medical staff  High workload | Breaches of patient confidentiality due to poor facilities; High workload, overcrowded due to lack of staff | Lack of personnel and infrastructure at OPCs | **Barrier** |
|  |  |  | Financial support for patients & providers  Material support at OPCs  Staff training | Financial support, incentives, and rewards for patients; Training, financial, personnel, and material support for providers | Training, material, personnel, and financial support | **Facilitator** |
| **Social influences** | Masculinity | Men should smoke; Smoking for socializing |  |  | Smoking is necessary for socializing and masculinity | **Barrier** |
|  | Family support  Provider support | Family can support; Family cannot help; Support from providers; “They can only advice.” | Provider support | Social and professional support | Social and provider support is crucial but not enough | **Facilitator** |
|  |  |  | Trust/relationship | Good relationships with patients; Trust in providers; Patients tend to listen to physicians/doctors | Trust and provider-patient relationship | **Facilitator** |
|  |  |  | Perceived providers’ role | Providers’ role is vital in providing TUT; | The perceived important role of providers and TUT at OPCs | **Facilitator** |
|  |  |  | Perceived importance of TUT | Providers perceived that TUT is essential for patients |  |  |
|  | Smoking stigma | Stigma/alienation from non-smokers |  |  | Alienation from non-smokers | **Barrier or Facilitator** |
| **Emotion** | Stress/depression | Stress coping; Smoke to ease depression/anxiety | Stress/depression | Stress/depression from stigma, unemployment, poverty, and family issues in patients | Smoking is “like a soulmate.” | **Barrier** |
|  | Health worries | Worries about the health of family and others; Worries about getting sick; Too ill to smoke | Fear of illness | Patients feared that they would get sick | Fear of illness and worries about the health of oneself and family | **Facilitator** |
| **Nature of the behaviours** | Addiction | Patients were too addicted to quit | Addiction | Patients were too addicted to quit | Nicotine addiction | **Barrier** |
|  | Strong cravings | Cravings were responsible for quitting difficulty | Strong cravings | Cravings were responsible for quitting difficulty |  | **Barrier** |
|  | Smoking habit | Smoking is a habitual behaviour | Smoking habit | Smoking is a habitual behaviour | Habitual smoking behaviour | **Barrier** |
| **Behavioural regulation** | Quit attempt | Patients tried to quit but failed |  |  | Previous failed quit attempt | **Facilitator** |
|  | Stimuli avoidance | Avoiding triggers can facilitate quitting |  |  | Avoidance of smoking environment and stimuli | **Facilitator** |
|  | Go outside to smoke | Outdoor smoking can facilitate quitting by reducing smoking frequency |  |  | Reduction in smoking | **Facilitator** |
|  | Reduction | Reduction in smoking can facilitate quitting |  |  |  | **Facilitator** |

*AIDS: Acquired immunodeficiency syndrome, NRT: Nicotine replacement therapy, OPC: outpatient clinic, TUT: TUT

The TDF *Social /professional role & identity* domain was merged with the *Social Influences* domain due to overlap in meaning in this context and is labelled *Social influences.*

**Table S2. TDF domains and associated main themes related to barriers and facilitators of smoking cessation in PLWH**

| **TDF domains** | | **Knowledge** | **Social Influences** | **Motivation & Goals** | **Environmental Context & Resources** |
| --- | --- | --- | --- | --- | --- |
| **Themes** | **Patient level** | Perceptions about the risk of tobacco use | Family support | Reasons of quitting | Environmental cues to smoke |
|  |  |  | Social norms and masculinity |  |  |
|  |  | Perceptions of smoking cessation treatment | Discrimination and stigma | Reasons for sustained tobacco use |  |
|  | **Provider level** | Healthcare providers lack knowledge about NRT | Therapeutic relationship between provider and patient |  |  |
|  | **System level (including organizational and policy level)** |  |  |  | Accessibility and perceived low cost of tobacco |
|  |  |  |  |  | Competing clinical demands impede tobacco use treatment |

*NRT: Nicotine replacement therapy, OPC: Outpatient clinic
